# Oral health and all-cause, cardiovascular disease, and respiratory mortality in older people in the UK and USA

**DOI:** 10.1038/s41598-021-95865-z

**Published:** 2021-08-12

**Authors:** Eftychia Kotronia, Heather Brown, A. Olia Papacosta, Lucy T. Lennon, Robert J. Weyant, Peter H. Whincup, S. Goya Wannamethee, Sheena E. Ramsay

**Affiliations:** 1 Population Health Sciences Institute, Newcastle University, The Baddiley-Clark Building, Richardson Road, Newcastle upon Tyne, NE2 4AX UK; 2 Department of Primary Care and Population Health, Institute of Epidemiology and Health Care, University College London, London, UK; 3 Department of Dental Public Health, School of Dental Medicine, University of Pittsburgh, Pittsburgh, PA USA; 4 Population Health Research Institute, St George’s University of London, London, UK

**Keywords:** Diseases, Risk factors

## Abstract

Preventing deterioration of oral health in older age can be crucial for survival. We aimed to examine associations of oral health problems with all-cause, cardiovascular disease (CVD), and respiratory mortality in older people. We used cohort data from the British Regional Health Study (BRHS) (N = 2147, 71–92 years), and the Health, Aging and Body Composition (HABC) Study (USA) (N = 3075, 71–80 years). Follow-up was 9 years (BRHS) and 15 years (HABC Study). Oral health comprised tooth loss, periodontal disease, dry mouth, and self-rated oral health. Cox regression was performed for all-cause mortality, competing risks for CVD mortality, and accelerated failure time models for respiratory mortality. In the BRHS, tooth loss was associated with all-cause mortality (hazard ratio (HR) = 1.59, 95% CI 1.09, 2.31). In the HABC Study, tooth loss, dry mouth, and having ≥ 3 oral problems were associated with all-cause mortality; periodontal disease was associated with increased CVD mortality (subdistribution hazard ratio (SHR) = 1.49, 95% CI 1.01, 2.20); tooth loss, and accumulation of oral problems were associated with high respiratory mortality (tooth loss, time ratio (TR) = 0.73, 95% CI 0.54, 0.98). Findings suggest that poor oral health is associated with mortality. Results highlight the importance of improving oral health to lengthen survival in older age.

## Introduction

Aging is characterized by an accumulation of chronic diseases and conditions, including poor oral health, which can influence quality of life and health^1,2^. Oral health problems, including tooth loss, periodontal disease, and dry mouth, accumulate throughout adult life and worsen with increasing age^3^. Poor dental health is associated with high levels of inflammation, poor diet quality, and conditions such as disability, diabetes, and increased risk of cardiovascular disease (CVD) and pneumonia^3–5^.

Furthermore, studies have suggested that poor oral health is associated with higher risk of mortality, including major causes of death such as CVD and respiratory diseases or infections. Tooth loss and periodontal disease have been reported to be associated with increased risks of all-cause, CVD and respiratory mortality in community-dwelling middle-aged and older people^6–13^. Additionally, hyposalivation (low production of saliva), was associated with greater risk of mortality in older men^14^. However, hyposalivation may not be indicative of self-perceived dryness of mouth (xerostomia), because people with normal production of saliva can report dryness of mouth^15^. Furthermore, poor self-rated oral health was found to be associated with increased risk of all-cause mortality in a population of middle-aged and older adults^16^.

Nevertheless, despite a number of studies examining associations between oral health and mortality, previous studies have reported inconsistent results for the associations between oral conditions and CVD and respiratory mortality in older people and more research is needed^17,18^. Moreover, tooth loss and periodontal disease have been the most commonly investigated oral health measures, with fewer studies examining subjective (self-reported) measures such as dry mouth, and self-rated oral health. Subjective measures are easier to assess, are established indicators of general oral health status and are associated with poor general health^19,20^; yet their associations with mortality remain unknown. Clarifying these associations can highlight the ways that burden of poor oral health influences survival. Therefore, we aim to examine the associations of several oral health problems with all-cause, CVD and respiratory mortality in two population-based studies of community-dwelling older people in the UK and USA.

## Methods

In this study we used data from the British Regional Heart Study (BRHS) in the UK and the Health, Aging and Body Composition (HABC) Study in the USA to examine associations of poor oral health with all-cause, CVD, and respiratory mortality. Including these two population-based studies allowed us to examine these associations in two comparable and complementary studies of older populations; the BRHS consists of white British men and the HABC Study comprises white and African American men and women in the USA.

### The British Regional Heart Study

This is an ongoing cohort study, which started in 1978–1980 and comprised 7735 White European British men aged 40–59 years. Individuals were recruited from 24 towns across the UK in 1978–80 and have been followed-up since^21^. For this study, data from the 30-year follow-up were used. The 30-year follow-up of the cohort was undertaken in 2010–2012 and 3137 surviving participants were invited to participate. In total, 2147 participants aged 71–92 years completed a postal questionnaire (68% response rate), and 1722 attended a physical and oral health examination (55% response rate) and had blood samples taken^21^. Oral examination was conducted by a trained nurse. Participants were followed-up from re-examination in 2010–2012 until June 2019. Details on causes of deaths were collected through the National Health Service Central Register (death certificates coded using International Classification of Diseases, ninth revision (ICD-9))^22^. The relevant local research ethics committees provided ethical approval. Written informed consent was obtained from individuals for their participation in the study, according to the Declaration of Helsinki.

### The Health, Aging and Body Composition Study

The HABC Study is a prospective population-based study investigating deterioration in physical function of older individuals and how changes in body composition influence health in older age. The cohort was initially examined in 1997–1998, where 3075 white and African-American males and females aged 70–79 years were recruited. Random selection of white participants was performed through Medicare, whereas African-American were selected through neighborhoods with a ZIP code around Memphis and Pittsburgh^23^. Individuals who were not able to walk 0.25 miles or climb 10 steps were excluded from the study. For this study, data from 2998 males and females aged 71–80 years from Year 2 were used (response rate = 97.5%). Measurements included oral health (n = 1975) and physical assessments, collection of blood samples and completion of questionnaires (n = 2998). Oral examination was conducted by a dental hygienist or periodontist. Participants were followed-up from re-examination in 1998–1999 until September 2014. Information on mortality was collected through death certificates. Causes of death were adjudicated according to review of medical records, proxy information and autopsy report by an independent HABC Study committee of gerontologists^24^. All participants provided written informed consent. Ethical approval was provided by several institutional review boards^23^.

### Oral health

In both studies, objective and subjective oral health problems were assessed through an oral examination and completion of questionnaires. Objective problems (oral examination) included count of natural teeth (tooth loss), and periodontal disease (loss of attachment and pocket depth). In both studies, a count of natural teeth was conducted during the oral examination. In the BRHS, periodontal measurements were carried out in six index teeth with a range of agreement between the examiner and the trainer from 89 to 95%, and a median κ index equal to 0.79^25^, whereas in the HABC Study a full mouth assessment took place with 90% agreement between examiners which was ascertained before the start of the dental examination^26^. Further details about the measurement of periodontal disease measures can be found elsewhere^25,26^. Number of natural teeth was classified as 5-level category (≥ 21, 15–20, 8–14,1–7 and 0 teeth). Periodontal pocket depth was classified for BRHS as > 20% sites affected > 3.5 mm, and for HABC. Study as > 20% sites affected ≥ 3 mm. Loss of attachment was categorized for BRHS as > 20% sites affected > 5.5 mm, and for HABC Study as > 20% sites affected ≥ 3 mm^27^. Subjective oral health problems (questionnaires) comprised self-rated oral health, dry mouth, difficulty eating due to mouth, teeth or dentures problems, sensitivity to hot/cold/sweets (BRHS), and limitation of food due to gum problems (HABC Study). In both studies, to assess self-rated oral health participants were asked to rate their oral health as excellent, good, fair, or poor. Then, participants were categorized as having either excellent/good or fair/poor self-rated oral health. In the BRHS, the Xerostomia Inventory Scale was used to measure dry mouth symptoms^28^. The XI comprises 11 items which include: mouth feels dry, difficulty eating dry foods, getting up at night to drink, mouth feels dry when eating a meal, sip liquids to aid swallowing food, sucking sweets to relieve dry mouth, difficulties swallowing certain foods, skin of face feels dry, eyes feel dry, lips feel dry, inside of nose feels dry^28^. Participants could choose the following responses: never, hardly ever, occasionally, fairly often or very often. According to the frequency of dry mouth symptoms (occasionally, fairly often or very often-dry mouth; never, hardly ever-no dry mouth symptoms), participants were classified as having 0, 1–2, and ≥ 3 dry mouth symptoms. In the HABC Study, one question was used to assess dry mouth (dry mouth when eating- either yes or no). A cumulative measure of oral health problems was also created which included, having 3 dry mouth symptoms, < 21 natural teeth, any difficulty eating, and sensitivity to hot, cold, or sweets in the BRHS, whereas in the HABC Study it consisted of dry mouth when eating, < 21 natural teeth, difficulty eating, and limitation of food because of gum problems^27^. The cumulative oral health problem variable was classified as 0, 1, 2, and 3 problems in both studies.

### Ascertainment of mortality

The outcomes in both studies were all-cause, CVD and respiratory mortality. In the BRHS, follow-up was defined as years from baseline/date of measurement to date of death or until the end of the follow-up period (June 2019). In the HABC Study, follow-up was defined as years from the study’s baseline/date of measurement to date of death or date of last contact.

### Covariates

In both studies, socioeconomic position measures, smoking, alcohol, physical activity, history of CVD and diabetes were self-reported through questionnaires^21,26^. Socioeconomic position was based on occupational social class (longest-held occupation at baseline) in the BRHS^25^, and on highest level of education attained in the HABC Study^26^. Body mass index (BMI) was calculated as body weight\height^2^ ratio, using measures from the physical examinations^29,30^. Diet quality (BRHS, Elderly Dietary Index (EDI); HABC Study, Healthy Eating Index (HEI)) was assessed through Food Frequency Questionnaires (FFQ)^22,31^. Inflammation (C-reactive protein (CRP), interleukin-6 (IL-6), fibrin d-dimer, high-sensitivity Troponin T (hsTnT)), blood pressure (mmHg), total blood cholesterol (mmol/l), triglycerides (mmol/l), HDL (mmol/l), respiratory rate (inspirations per 30 s), forced expiratory volume (FEV_1_), and forced vital capacity (FVC) were obtained from blood samples and physical examinations^4^. In both studies, information on medications causing xerostomia was also available from questionnaires at baseline.

### Statistical analysis

Survival analyses were performed for all cause, CVD, and respiratory mortality. For all-cause mortality analysis we performed Cox Proportional Hazards regression and obtained effect estimates as hazard ratios (HR) and 95% confidence intervals (CI). For CVD mortality we performed competing risks analysis, to account for the competing risk of non-CVD deaths^32^. We used the Fine-Grey subdistribution hazard model, which estimates the cumulative incidence function in the presence of competing risks^33^. The use of cumulative incidence function provides more reliable estimates^32^. Not taking account of competing risks can lead to overestimation of the incidence function^32^. Results are presented as subdistribution hazard ratios (SHR) with 95%CI.

The proportional hazards assumption, hazard function is constant over time, was violated for respiratory mortality, and therefore, we performed an Accelerated Failure Time (AFT) regression. Under the AFT assumption, an acceleration factor is added to the hazard function of the model^34^. This model is similar to a linear regression, however the errors do not follow a normal distribution^34^. After conducting diagnostic tests, we chose to model our data according to the Weibull survival distribution^35,36^. To ease interpretation of results, we will present the effect estimates as time ratios (TR), which is the exponentiated coefficient, with 95% CI. A times ratio lower than 1 indicates that the exposure is harmful and associated with decreased survival.

In the BRHS, all-cause and CVD mortality models were adjusted for age, social class, smoking, alcohol, physical activity, history of CVD and diabetes, BMI, diet quality, hypertension, triglycerides, HDL, CRP, IL-6, fibrin d-dimer and hsTnT. For respiratory mortality, the model was additionally adjusted for FEV_1_/FVC ratio. In the HABC study, models were adjusted for age, gender, race, smoking, alcohol, physical activity, history of CVD and diabetes, diet quality, hypertension, total blood cholesterol, CRP, and IL-6. For respiratory mortality, the model was adjusted further for respiratory rate. All analyses were performed in STATA 15.1 (Stata Corp LLC, College Station, Texas).

## Results

Population characteristics at baseline are presented in Table 1.

**Table 1:** Population characteristics in the British Regional Heart Study (BRHS) and the Health, Aging, and Body Composition (HABC) Study.

BRHS (n = 2147)		HABC study (n = 2998)	
**Age (years), median (IQR)**	77.7 (74.8–82.1)	**Age (years), median (IQR)**	74 (72–77)
		**Gender, n (%)**	
		Male	1491 (48)
		Female	1584 (52)
		**Race, n (%)**	
		White	1794 (58)
		African American	1281 (42)
**Social class, n (%)**		**Education, n (%)**	
Nonmanual	1081 (52)	Less than high school	775 (25)
Manual	1003 (48)	High school graduate	1000 (33)
		Postsecondary	1292 (42)
**Smoking, n (%)**		Smoking*, n (%)	
Never	768 (36)	Never	1348 (44)
Current smoker	91 (4)	Current smoker	318 (10)
Ex-smoker	1275 (60)	Ex-smoker	1401 (46)
**Alcohol, n (%)**		Alcohol*, n (%)	
Daily	757 (36)	No consumption past year	1546 (50)
Occasionally	307 (15)	1–7 times per week	655 (21)
None	292 (14)	More than 1 per day	227 (7)
**Physical activity, n (%)**		Physical activity* (kcal/kg/week), median (IQR)	64.5 (38.3–106.5)
Inactive	405 (20)		
Occasional	475 (24)		
Moderate	278 (14)		
**History of cardiovascular disease, n (%)**	500 (24)	**History of cardiovascular disease, n (%)**	106 (4)
**History of diabetes, n (%)**	321 (15)	**History of diabetes, n (%)**	142 (5)
**Body mass index, n (%)**		**Body mass index, n (%)**	
Normal	486 (29)	Normal	963 (34)
Overweight	875 (51)	Overweight	1192 (42)
Obese	343 (20)	Obese	673 (24)
**EDI score, median (IQR)**	25 (23–27)	**HEI score, median (IQR)**	71 (61–79)
**FEV1/FVC ratio**	0.75 (0.70–0.80)	**Respiration rate (insp /30 s), median (IQR)**	9 (8–10)
**Hypertension, n (%)**	473 (28)	**Hypertension, n (%)**	765 (25)
**Triglycerides (mmol/l), median (IQR)**	1.14 (0.84–1.54)	**Total blood cholesterol (mg/dl), median (IQR)**	204 (179–229)
**HDL (mmol/l), median (IQR)**	1.40 (1.16–1.68)		
**CRP (ug/ml), median (IQR)**	1.28 (0.61–2.93)	**CRP (ug/ml), median (IQR)**	2.97 (1.25–6.44)
**IL-6 (pg/ml), median (IQR)**	2.85 (1.82–4.68)	**Il-6 (pg/ml), median (IQR)**	2.40 (1.52–4.04)
**Fibrin d-dimer ng/ml),** **median (IQR)**	216.53 (153.94–328.11)		
**High sensitivity Troponin T (ng/l), median (IQR)**	11.34 (7.26–16.92)		

*EDI* elderly dietary index, *HEI* healthy eating index.

*Measured at Year 1 (1997–1998).

In the BRHS, median age was 77.7 years, 48% were manual workers, 4% were current smokers and 36% consumed alcohol daily. Additionally, 24% had a history of CVD, 51% were overweight and 28% had hypertension. Furthermore, 20% of the participants had no natural teeth, 24% had > 20% of sites with loss of attachment, 35% reported fair/poor self-rated oral health, 62.5% had at least 1 dry mouth symptom and 36% had at least 2 oral health problems. In the HABC Study, median age was 74 years, 52% of the participants were female, 42% African American and 42% had completed postsecondary education. Furthermore, 10% were current smokers, 7% consumed alcohol more than once per day, 4% had history of CVD, 42% were overweight, and 25% had hypertension. For oral health problems, 10.5% had no natural teeth, 64% had > 20% of sites with loss of attachment, 30.5% reported fair/poor self-rated oral health, 4% had dry mouth symptoms when eating, and 22% had at least 2 oral health problems.

### Oral health problems and the association with all-cause mortality

In the BRHS median survival time was 7.75 years and in the HABC Study 12.6 years. Hazard ratios (HR) and 95% CI for the association between oral health problems and all-cause mortality in the BRHS and HABC Study are presented in Table 2. In the BRHS, partial tooth loss (1–7 teeth vs. ≥ 21 teeth) was associated with increased risk of all-cause mortality after adjustment for confounders (HR = 1.59, 95% CI: 1.09–2.31). Periodontal disease, having at least 2 dry mouth symptoms and cumulative oral health problems were associated with greater risk of mortality in the age-adjusted models, but associations were attenuated after adjustment for confounders. In the HABC Study, partial (1–7 teeth vs. ≥ 21) and complete tooth loss (0 teeth vs. ≥ 21) were associated with greater risk of all-cause mortality in the fully adjusted models (HR = 1.33, 95% CI: 1.06–1.65; HR = 1.33, 95% CI: 1.08–1.63, respectively). Additionally, dry mouth and cumulative oral health problems (≥ 3 oral problems vs. 0) were associated with increased risk of all-cause mortality in the fully adjusted models (HR = 1.46, 95% CI: 1.15–1.84; HR = 1.27, 95%CI: 1.04–1.55, respectively). The association between dry mouth and all-cause mortality did not change significantly after adjusting for medications causing xerostomia.

**Table 2:** Hazard ratios (HR) and 95% confidence intervals (CI) for the association between oral health problems and risk of all-cause mortality in older people in the BRHS after 9 years of follow-up and in the HABC Study after 15 years of follow-up.

	BRHS			HABC study	
	All-cause mortality			All-cause mortality	
	Age adjusted HR (95% CI)	Fully adjusted* HR (95% CI)		Age adjusted HR (95% CI)	Fully adjusted^†^ HR (95% CI)
**Tooth loss**			**Tooth loss**		
≥ 21 teeth (n = 594, 36%)	1.00	1.00	≥ 21 teeth (n = 943, 48%)	1.00	1.00
15–20 teeth (n = 340, 20%)	1.14 (0.88, 1.47)	0.97 (0.71, 1.32)	15–20 teeth (n = 357, 18%)	1.11 (0.95, 1.30)	0.98 (0.83, 1.16)
8–14 teeth (n = 265, 16%)	1.40 (1.08, 1.82)	1.06 (0.76, 1.47)	8–14 teeth (n = 282, 14%)	1.22 (1.03, 1.44)	1.08 (0.90, 1.30)
1–7 teeth (n = 123, 7%)	1.71 (1.26, 2.32)	1.59 (1.09, 2.31)	1–7 teeth (n = 184, 9%)	1.48 (1.21, 1.81)	1.33 (1.06, 1.65)
0 teeth (n = 338, 20%)	1.71 (1.36, 2.15)	1.16 (0.85, 1.57)	0 teeth (n = 208, 10%)	1.47 (1.23, 1.76)	1.33 (1.08, 1.63)
**Periodontal disease**^||^**(% of sites with loss of attachment > 5.5 mm)**			**Periodontal disease**^||^**(% of sites with pocket depth ≥ 3 mm)**		
≤ 20% (n = 943, 76%)	1.00	1.00	≤ 20% (n = 509, 45%)	1.00	1.00
> 20% (n = 303, 24%)	1.45 (1.16, 1.81)	1.24 (0.94, 1.63)	> 20% (n = 627, 55%)	1.17 (1.00, 1.48)	1.09 (0.92, 1.29)
**Self-rated oral health**			**Self-rated oral health**		
Good/excellent (n = 1320, 65%)	1.00	1.00	Good/excellent (n = 1889, 70%)	1.00	1.00
Fair/poor (n = 719, 35%)	1.15 (0.99, 1.33)	1.10 (0.89, 1.36)	Fair/poor (n = 829, 30%)	1.11 (1.00, 1.23)	0.99 (0.89, 1.11)
**Dry mouth symptoms**			**Dry mouth**		
0 (n = 762, 37%)	1.00	1.00	No (n = 2612, 96%)	1.00	1.00
1–2 (n = 666, 33%)	1.07 (0.89, 1.28)	1.10 (0.85, 1.42)	Yes (n = 107, 4%)	1.52 (1.23, 1.90)	1.46 (1.15, 1.84)
≥ 3 (n = 606, 30%)	1.32 (1.11, 1.56)	1.22 (0.94, 1.57)			
**Cumulative oral health problems**^‡^			**Cumulative oral health problems**^**§**^		
0 (n = 340, 16%)	1.00	1.00	0 (n = 778, 28%)	1.00	1.00
1 (n = 1041, 49%)	1.48 (1.17, 1.88)	1.23 (0.90, 1.67)	1 (n = 1367, 49%)	1.30 (1.16, 1.45)	1.15 (1.02, 1.30)
2 (n = 540, 25%)	1.74 (1.35, 2.23)	1.29 (0.92, 1.82)	2 (n = 394, 14%)	1.25 (1.07, 1.46)	1.08 (0.92, 1.28)
≥ 3 (n = 226, 10%)	1.79 (1.34, 2.38)	1.32 (0.88, 1.97)	≥ 3 (n = 223, 8%)	1.45 (1.21, 1.74)	1.27 (1.04, 1.55)

*Age, social class, smoking, alcohol, physical activity, history of CVD and diabetes, BMI, EDI score, hypertension, triglycerides, HDL, CRP, IL-6, fibrin d-dimer, hsTnT.

^†^Age, gender, race, education, smoking, alcohol, physical activity, history of CVD and diabetes, BMI, HEI score, hypertension, total blood cholesterol, CRP, IL-6.

^‡^Includes < 21 teeth, difficulty eating, symptoms of dry mouth, and sensitivity to hot/cold/sweet.

^§^Includes < 21 teeth, difficulty eating, dry mouth when eating, and limit of food due to gum problems.

^||^Only measures of periodontal disease with positive associations included.

### Oral health problems and the association with CVD mortality

Table 3 presents the results from the competing risks analysis for the association of oral health problems with CVD mortality in both studies. In the BRHS, partial tooth loss was associated with greater relative incidence of CVD death after 9 years of follow-up (1–7 teeth vs. ≥ 21 teeth, SHR = 2.11, 95% CI: 1.27–3.51) in the age adjusted model but were attenuated and did not remain after adjustment. Additionally, a 50% increase (non-significant) in incidence of CVD mortality was reported for periodontal disease (SHR = 1.50, 95% CI 0.93–2.40), in the fully adjusted model. Likewise, in the HABC Study, periodontal disease was associated with a significant increase in relative incidence of CVD mortality by 49% after 15 years of follow up (SHR = 1.49, 95% 1.01–2.20) in the fully adjusted model.

**Table 3:** Competing risk analysis (subdistribution hazard ratios (SHR) and 95% confidence intervals (CI)) for the association between oral health problems and risk of CVD mortality in older people in the BRHS after 9 years of follow-up and in the HABC Study after 15 years of follow-up.

	BRHS CVD mortality			HABC study CVD mortality	
	Age adjusted SHR (95% CI)	Fully adjusted* SHR (95% CI)		Age adjusted SHR (95% CI)	Fully adjusted^†^ SHR (95% CI)
**Tooth loss**			**Tooth loss**		
≥ 21 teeth (n = 594, 36%)	1.00	1.00	≥ 21 teeth (n = 943, 48%)	1.00	1.00
15–20 teeth (n = 340, 20%)	1.14 (0.72, 1.80)	0.99 (0.56, 1.73)	15–20 teeth (n = 357, 18%)	0.91 (0.65, 1.29)	0.91 (0.62, 1.32)
8–14 teeth (n = 265, 16%)	1.92 (1.24, 2.95)	1.53 (0.86, 2.71)	8–14 teeth (n = 282, 14%)	1.31 (0.93, 1.83)	1.25 (0.85, 1.82)
1–7 teeth (n = 123, 7%)	2.11 (1.27, 3.51)	1.67 (0.82, 3.40)	1–7 teeth (n = 184, 9%)	0.93 (0.59, 1.46)	0.87 (0.52, 1.47)
0 teeth (n = 338, 20%)	1.33 (0.86, 2.07)	1.04 (0.59, 1.85)	0 teeth (n = 208, 10%)	1.37 (0.95, 1.98)	1.46 (0.95, 2.26)
**Periodontal disease**^**||**^** (% of sites with pocket depth > 3.5 mm)**			**Periodontal disease**^||^**(% of sites with pocket depth ≥ 3 mm)**		
≤ 20% (n = 880, 71%)	1.00	1.00	≤ 20% (n = 509, 45%)	1.00	1.00
> 20% (n = 365, 29%)	1.27 (0.88, 1.85)	1.50 (0.93, 2.40)	> 20% (n = 627, 55%)	1.54 (1.08, 2.19)	1.49 (1.01, 2.20)
**Self-rated oral health**			**Self-rated oral health**		
Good/excellent (n = 1320, 65%)	1.00	1.00	Good/excellent (n = 1889, 70%)	1.00	1.00
Fair/poor (n = 719, 35%)	1.21 (0.94, 1.57)	1.14 (0.77, 1.69)	Fair/poor (n = 829, 30%)	1.09 (0.88, 1.35)	1.12 (0.90, 1.40)
**Dry mouth symptoms**			**Dry mouth**		
0 (n = 762, 37%)	1.00	1.00	No (n = 2612, 96%)	1.00	1.00
1–2 (n = 666, 33%)	1.14 (0.83, 1.56)	1.12 (0.69, 1.81)	Yes (n = 107, 4%)	1.32 (0.84, 2.07)	1.39 (0.86, 2.23)
≥ 3 (n = 606, 30%)	1.33 (0.97, 1.80)	1.31 (0.81, 2.12)			
**Cumulative oral health problems**^‡^			**Cumulative oral health problems**^§^		
0 (n = 340, 16%)	1.00	1.00	0 (n = 778, 28%)	1.00	1.00
1 (n = 1041, 49%)	0.98 (0.66, 1.46)	0.68 (0.40, 1.18)	1 (n = 1367, 49%)	1.18 (0.93, 1.50)	1.16 (0.90, 1.51)
2 (n = 540, 25%)	1.49 (0.98, 2.26)	1.10 (0.62, 1.94)	2 (n = 394, 14%)	1.23 (0.90, 1.68)	1.27 (0.91, 1.78)
≥ 3 (n = 226, 10%)	1.40 (0.86, 2.28)	0.97 (0.50, 1.92)	≥ 3 (n = 223, 8%)	1.05 (0.70, 1.58)	1.10 (0.70, 1.71)

*Age, social class, smoking, alcohol, physical activity, history of CVD and diabetes, BMI, EDI score, hypertension, triglycerides, HDL, CRP, IL-6, fibrin d-dimer, hsTnT.

^†^Age, gender, race, education, smoking, alcohol, physical activity, history of CVD and diabetes, BMI, HEI score, hypertension, total blood cholesterol, CRP, IL-6.

^‡^Includes < 21 teeth, difficulty eating, symptoms of dry mouth, and sensitivity to hot/cold/sweet.

^§^Includes < 21 teeth, difficulty eating, dry mouth when eating, and limit of food due to gum problems.

^||^Only measures of periodontal disease with positive associations included.

### Oral health problems and the association with respiratory mortality

Associations between oral health problems and respiratory mortality in the BRHS and HABC Study are presented in Table 4. In the BRHS, only in the age adjusted model, partial and complete tooth loss when compared to ≥ 21 teeth (functional dentition) were associated with a 54% (95% CI 0.27–0.78) and 44% (95% CI 0.36–0.87) shorter survival due to respiratory causes, respectively. In the HABC Study, both partial and complete tooth loss were associated with shorter survival due to respiratory causes after adjustment for confounders (fully adjusted, TR = 0.73, 95%CI 0.55–0.57; TR = 0.73, 95% CI 0.54–0.98, respectively). Additionally, having 2 oral health problems was associated with a 37% shorter survival when compared to no oral health problems after adjustment for confounders.

**Table 4:** Accelerated failure time analysis (time ratios (TR) and 95% confidence intervals (CI)) for the association between oral health problems and respiratory mortality in older people in the BRHS after 9 years of follow-up and the HABC Study after 15 years of follow-up.

	BRHS			HABC study	
	Respiratory mortality			Respiratory mortality	
	Age adjusted TR (95% CI)	Fully adjusted* TR (95% CI)		Age adjusted TR (95% CI)	Fully adjusted^†^ TR (95% CI)
**Tooth loss**			**Tooth loss**		
≥ 21 teeth (n = 594, 36%)	1.00	1.00	≥ 21 teeth (n = 943, 48%)	1.00	1.00
15–20 teeth (n = 340, 20%)	0.76 (0.47, 1.23)	0.89 (0.53, 1.50)	15–20 teeth (n = 357, 18%)	0.87 (0.69, 1.11)	0.95 (0.74, 1.21)
8–14 teeth (n = 265, 16%)	0.72 (0.43, 1.18)	1.15 (0.60, 2.22)	8–14 teeth (n = 282, 14%)	0.86 (0.66, 1.12)	0.88 (0.68, 1.14)
1–7 teeth (n = 123, 7%)	0.46 (0.27, 0.78)	0.85 (0.41, 1.75)	1–7 teeth (n = 184, 9%)	0.76 (0.57, 1.02)	0.73 (0.54, 0.98)
0 teeth (n = 338, 20%)	0.56 (0.36, 0.87)	0.72 (0.44, 1.19)	0 teeth (n = 208, 10%)	0.74 (0.56, 0.97)	0.73 (0.55, 0.97)
**Periodontal disease**^||^**(% of sites with loss of attachment > 5.5 mm)**			**Periodontal disease**^||^**(% of sites with loss of attachment ≥ 3 mm)**		
≤ 20% (n = 943, 76%)	1.00	1.00	≤ 20% (n = 412, 36%)	1.00	1.00
> 20% (n = 303, 24%)	0.85 (0.59, 1.23)	1.04 (0.65, 1.64)	> 20% (n = 721, 64%)	0.83 (0.65, 1.07)	0.90 (0.70, 1.16)
**Self-rated oral health**			**Self-rated oral health**		
Good/excellent (n = 1320, 65%)	1.00	1.00	Good/excellent (n = 1889, 70%)	1.00	1.00
Fair/poor (n = 719, 35%)	0.85 (0.66, 1.09)	1.02 (0.71, 1.45)	Fair/poor (n = 829, 30%)	0.87 (0.75, 1.02)	0.97 (0.83, 1.14)
**Dry mouth symptoms**			**Dry mouth**		
0 (n = 762, 37%)	1.00	1.00	No (n = 2612, 96%)	1.00	1.00
1–2 (n = 666, 33%)	0.89 (0.66, 1.21)	1.05 (0.71, 1.55)	Yes (n = 107, 4%)	0.71 (0.52, 0.96)	0.75 (0.55, 1.02)
≥ 3 (n = 606, 30%)	0.84 (0.62, 1.12)	1.24 (0.81, 1.90)			
**Cumulative oral health problems**^‡^			**Cumulative oral health problems**^§^		
0 (n = 340, 16%)	1.00	1.00	0 (n = 778, 28%)	1.00	1.00
1 (n = 1041, 49%)	0.61 (0.39, 0.96)	0.93 (0.56, 1.54)	1 (n = 1367, 49%)	0.79 (0.65, 0.96)	0.85 (0.70, 1.03)
2 (n = 540, 25%)	0.65 (0.40, 1.04)	1.11 (0.62, 1.96)	2 (n = 394, 14%)	0.70 (0.55, 0.90)	0.77 (0.61, 0.97)
≥ 3 (n = 226, 10%)	0.65 (0.38, 1.12)	1.38 (0.64, 3.00)	≥ 3 (n = 223, 8%)	0.64 (0.49, 0.84)	0.76 (0.58, 1.01)

*Age, social class, smoking, alcohol, physical activity, history of CVD and diabetes, BMI, EDI score, FEV1/FVC ratio, hypertension, triglycerides, HDL, CRP, IL-6, fibrin d-dimer, hsTnT.

^†^Age, gender, race, education, smoking, alcohol, physical activity, history of CVD and diabetes, BMI, HEI score, respiration rate, hypertension, total blood cholesterol, CRP, IL-6.

^‡^Includes < 21 teeth, difficulty eating, symptoms of dry mouth, and sensitivity to hot/cold/sweet.

^§^Includes < 21 teeth, difficulty eating, dry mouth when eating, and limit of food due to gum problems. ^||^Only measures of periodontal disease with positive associations included.

## Discussion

In this prospective study of older population-based cohorts in the UK and USA, oral health problems were associated with greater risk of all-cause mortality after adjusting for chronic diseases, biological, and behavioral factors in both studies. Additionally, in the HABC Study, poor oral health was associated with greater risk of CVD and respiratory mortality. Particularly, tooth loss, dry mouth and accumulation of oral health problems were associated with all-cause mortality. Furthermore, periodontal disease was associated with increased incidence of CVD mortality after taking account of competing risk of non-CVD deaths. Finally, tooth loss and cumulative oral health problems were associated with high respiratory mortality after adjusting for confounders.

In both studies, partial tooth loss was associated with all-cause mortality. Previous studies have provided inconsistent results, with few of them investigating associations for both complete and partial tooth loss (measured objectively through number of remaining natural teeth) and risk of mortality^7–10,37–40^. In this study, tooth loss was assessed objectively, and associations of both complete and partial tooth loss with greater risk of all-cause mortality were established. Potential pathways linking tooth loss with mortality include compromised diet and increased inflammation. However, we did not examine potential mediating effects and only adjusted regression models for diet quality and inflammation in both studies. First, it is possible that tooth loss-related chewing problems which is the main cause of food avoidance and therefore compromised diet may explain the association between tooth loss and mortality^41^. Second, tooth loss can be a result of periodontal disease and dental caries, with both conditions being associated with the dental biofilm of infected teeth, and therefore associated with increased levels of inflammatory markers^42,43^. Chronic oral inflammation, and particularly bacteria from the mouth, can travel through circulation to other parts of the body and may influence the development or severity of chronic diseases^4^. Tooth loss as a reflection of accumulating chronic conditions and declining health can contribute to the increased risk of mortality through its interaction with co-morbidities^5,44^.

Furthermore, we observed associations between dry mouth and accumulation of oral health problems with all-cause mortality in the HABC Study. This is one of the first studies examining the association of dry mouth and cumulative oral health problems with risk of all-cause mortality in predominantly community-dwelling older populations. A previous study, reported an association between low production of saliva (objective measure of dry mouth) and greater risk of all-cause mortality in older people^14^. Perception of dry mouth is common in older populations. It is often a consequence of (a) medications use (or polypharmacy) for chronic diseases such as diabetes, (b) systemic diseases such as chronic inflammatory autoimmune diseases, neurologic disorders^19,28^, and can be associated with increased risk of hypertension^45^. Nevertheless, the association observed in our study was independent of hypertension and medication use. Finally, no associations were observed for fair/poor self-reported oral health and risk of all-cause mortality in both studies, which is in contrast with one previous study conducted in a population ≥ 45 years^16^. However, this previous study included both middle-aged and older individuals.

Periodontal disease was associated with increased risk of CVD mortality in the HABC Study while considering the competing risk of non-CVD deaths, in the fully adjusted model. This finding is in accordance with one previous study conducted in an older Taiwanese population^11^. However, none of the identified studies have performed competing risks analysis to investigate the association between periodontal disease and risk of CVD mortality. The association observed in our study persisted even after adjusting for inflammatory markers. However, we adjusted for levels of inflammation measured at one time point which may not reflect chronic inflammation. Periodontal disease is a chronic inflammatory oral disease potentially contributing to chronic inflammation^46^. Periodontal disease could also be associated with thrombus formation, atherosclerosis and stroke^47^. It has been suggested that the most frequent bacteria found in atherosclerotic plaques come from the oral microflora^48,49^. Interestingly, effect estimates for the association between periodontal disease and CVD mortality in the BRHS were similar, although non-significant, to those observed in the HABC Study. This could be due to the smaller sample in the BRHS than the HABC Study. Nevertheless, these results suggest that older individuals with periodontal disease may be in greater risk of CVD mortality. Furthermore, this was one of the first studies to examine associations of self-rated oral health, dry mouth, and accumulation of oral health problems with risk of CVD mortality in community-dwelling older people. However, we did not observe associations between subjective oral health problems (self-rated oral health, dry mouth) and risk of CVD mortality.

In the HABC study, partial and complete tooth loss were associated with increased respiratory mortality after adjustment for confounders. This finding was supported by two previous studies in older Japanese populations^6,50^. It has been suggested that eating difficulties along with tooth loss resulted in increased risk of respiratory mortality^6^. In addition, it is possible that frequency of tooth brushing could influence the reported association, because oral hygiene is a factor which can influence tooth retention and respiratory mortality^51^. Tooth loss could also be a product of chronic periodontal disease^52^, where bacteria from periodontal lesions are circulated to the lungs causing aspiration pneumonia^53^. However, we did not observe any associations between periodontal disease and respiratory mortality. It is possible that in both populations included in our study healthier teeth remained and were assessed for periodontal disease. Furthermore, since we did not have a prior history of oral health it is hard to assess the influence of previous chronic periodontal disease on tooth loss. Furthermore, another potential pathway could be through low levels of vitamin D, which have been associated with both increased tooth loss as well as respiratory mortality^54,55^. We did not observe any associations between subjective oral health problems and respiratory mortality in both studies. One previous study reported an association between dry mouth and greater risk of respiratory mortality, however this study was conducted in a population of middle-aged and older individuals^56^. It is possible that self-reported oral health problems in middle age may have a greater impact on life expectancy than dental problems in older age.

This is one of the first studies performing competing risks analysis to examine the association between oral health problems and cardiovascular mortality while taking account of the competing risk of non-CVD deaths in older people, which provides robust results about the associations with CVD mortality. Additionally, this study adds to the limited evidence on the associations between subjective oral health problems and risk of all-cause mortality, as well as cause-specific mortality^18^. Our study has some limitations. Some oral health problems (i.e., periodontal disease, dry mouth) were measured differently in the two studies and the follow-up period was shorter in the BRHS than the HABC Study, therefore potentially contributing to differences in the observed associations between the two studies. Additionally, in the BRHS, there was a small number of people with oral health problems who died from CVD or respiratory mortality, leading to a small sample size. This may have also accounted for non-significant results in the BRHS, as well as differences in effect estimates observed in associations in the two studies. Moreover, in both studies, the majority of reported associations between poor oral health and risk of all-cause and cause-specific mortality were observed for objective oral health problems, such as tooth loss and periodontal disease. For subjective dental problems, only dry mouth was associated with all-cause mortality after full adjustment in the HABC Study. This raises the issue of the validity of self-reported measures of oral health as predictors of mortality in older populations. In addition, information on periodontal treatment during follow-up period was not available, and therefore we were unable to examine its potential effect on reported associations between periodontal disease and risk of mortality. In addition, details about occlusion of teeth were not available and therefore we were not able to investigate the associations between this variable and risk of mortality. In both studies, it is highly possible that healthier individuals were able to answer questionnaires and attend the examinations and as a result the observed associations may be underestimated. Furthermore, we were unable to test for the competing risk of cardiovascular disease in the analysis for respiratory mortality. In both studies, to account for various factors influencing associations between poor oral health and mortality, we adjusted for several confounders. However, it is possible that we may not have included all potential confounders, including factors such as social support and cognitive status which can influence the magnitude and significance of associations. Finally, our findings may not be representative of the general populations of the UK and USA. The BRHS consisted of white males, whereas the HABC Study comprised a subpopulation of older people in the US (White and African American men and women from Pittsburgh and Memphis).

In conclusion, different objective and subjective oral health problems were associated with all-cause mortality in both studies of older populations in the UK and USA. Some oral health markers were also associated with CVD and respiratory mortality. Our findings suggest that older individuals with poor oral health may have a reduced life expectancy, independent of behavioral and biological factors. Oral health conditions may be modifiable risk factors in improving disease prognosis and survival in community-dwelling older people. More studies are needed examining the influence of (a) other objectively assessed dental diseases including apical periodontal disease and temporomandibular joint disorders and (b) self-reported measures of oral health on the risk of mortality in older people, to validate their role as potential predictors. Additionally, further research is needed on whether oral health improvement and dental treatment result in reducing adverse cardiovascular and respiratory outcomes in older populations.

## Data Availability

Data used in this study can be available upon request.

## References

[1] Kassebaum NJ (2017). Global, regional, and national prevalence, incidence, and disability-adjusted life years for oral conditions for 195 countries, 1990–2015: A systematic analysis for the global burden of diseases, injuries, and risk factors. J. Dent. Res..

[2] Nunes BP, Flores TR, Mielke GI, Thumé E, Facchini LA (2016). Multimorbidity and mortality in older adults: A systematic review and meta-analysis. Arch. Gerontol. Geriatr..

[3] Scannapieco FA, Cantos A (2016). Oral inflammation and infection, and chronic medical diseases: Implications for the elderly. Periodontol..

[4] Kotronia E (2020). Poor oral health and inflammatory, hemostatic, and cardiac biomarkers in older age: Results from two studies in the UK and USA. J. Gerontol. Ser. A.

[5] Friedman PK, Lamster IB (2016). Tooth loss as a predictor of shortened longevity: exploring the hypothesis. Periodontol..

[6] Aida J (2011). Oral health and cancer, cardiovascular, and respiratory mortality of Japanese. J. Dent. Res..

[7] Ansai T (2010). Relationship between tooth loss and mortality in 80-year-old Japanese community-dwelling subjects. BMC Public Health.

[8] Goto Y (2020). Number of teeth and all-cause and cancer mortality in a Japanese community: The Takayama study. J. Epidemiol..

[9] Hirotomi T, Yoshihara A, Ogawa H, Miyazaki H (2015). Number of teeth and 5-year mortality in an elderly population. Commun. Dent. Oral Epidemiol..

[10] Schwahn C (2013). Missing, unreplaced teeth and risk of all-cause and cardiovascular mortality. Int. J. Cardiol..

[11] Chen Y-T (2015). Periodontal disease and risks of kidney function decline and mortality in older people: A community-based cohort study. Am. J. Kidney Dis..

[12] Linden GJ (2012). All-cause mortality and periodontitis in 60–70-year-old men: A prospective cohort study. J. Clin. Periodontol..

[13] Qian Y (2020). Periodontitis increases the risk of respiratory disease mortality in older patients. Exp. Gerontol..

[14] Iwasaki M (2018). Hyposalivation and 10-year all-cause mortality in an elderly Japanese population. Gerodontology.

[15] Wiener RC (2010). Hyposalivation and xerostomia in dentate older adults. J. Am. Dent. Assoc..

[16] Joshy G, Arora M, Korda RJ, Chalmers J, Banks E (2016). Is poor oral health a risk marker for incident cardiovascular disease hospitalisation and all-cause mortality? Findings from 172 630 participants from the prospective 45 and up study. BMJ Open.

[17] Koka S, Gupta A (2018). Association between missing tooth count and mortality: A systematic review. J. Prosthodont. Res..

[18] Peng J (2019). The relationship between tooth loss and mortality from all causes, cardiovascular diseases, and coronary heart disease in the general population: Systematic review and dose–response meta-analysis of prospective cohort studies. Biosci. Rep..

[19] Han P, Suarez-Durall P, Mulligan R (2015). Dry mouth: A critical topic for older adult patients. J. Prosthodont. Res..

[20] Locker D (1997). Clinical correlates of changes in self-perceived oral health in older adults. Commun. Dent. Oral Epidemiol..

[21] Lennon LT (2015). Cohort profile update: The British Regional Heart Study 1978–2014: 35 years follow-up of cardiovascular disease and ageing. Int. J. Epidemiol..

[22] Atkins JL (2014). High diet quality is associated with a lower risk of cardiovascular disease and all-cause mortality in older men. J. Nutr..

[23] Stewart R (2013). Adverse oral health and cognitive decline: The health, aging and body composition study. J. Am. Geriatr. Soc..

[24] Cesari M (2012). Oxidative damage, platelet activation, and inflammation to predict mobility disability and mortality in older persons: Results from the health aging and body composition study. J Gerontol. Ser. A Biol. Sci. Med. Sci..

[25] Ramsay SE (2015). Burden of poor oral health in older age: findings from a population-based study of older British men. BMJ Open.

[26] Weyant RJ (2004). Periodontal disease and weight loss in older adults. J. Am. Geriatr. Soc..

[27] Kotronia E (2019). Oral health, disability and physical function: Results from studies of older people in the United Kingdom and United States of America. J. Am. Med. Dir. Assoc..

[28] Thomson WM, Chalmers JM, Spencer AJ, Williams SM (1999). The Xerostomia Inventory: A multi-item approach to measuring dry mouth. Commun. Dent. Health.

[29] Health ABCS (2007). Body mass index and serum leptin concentration independently estimate percentage body fat in older adults. Am. J. Clin. Nutr..

[30] Parsons TJ (2017). Physical activity, sedentary behavior, and inflammatory and hemostatic markers in men. Med. Sci. Sports Exerc..

[31] Hengeveld LM (2018). Prospective associations of poor diet quality with long-term incidence of protein-energy malnutrition in community-dwelling older adults: the Health, Aging, and Body Composition (Health ABC) Study. Am. J. Clin. Nutr..

[32] Austin PC, Lee DS, Fine JP (2016). Introduction to the analysis of survival data in the presence of competing risks. Circulation.

[33] Fine JP, Gray RJ (1999). A proportional hazards model for the subdistribution of a competing risk. J. Am. Stat. Assoc..

[34] Coppini DV, Bowtell PA, Weng C, Young PJ, Sönksen PH (2000). Showing neuropathy is related to increased mortality in diabetic patients- A survival analysis using an accelerated failure time model. J. Clin. Epidemiol..

[35] Collett D (2014). Modelling Survival Data in Medical Research.

[36] Wei LJ (1992). The accelerated failure time model: A useful alternative to the cox regression model in survival analysis. Stat. Med..

[37] Ajwani S, Mattila KJ, Narhi TO, Tilvis RS, Ainamo A (2003). Oral health status, C-reactive protein and mortality – A 10 year follow-up study. Gerodontology.

[38] Hämäläinen P, Meurman JH, Keskinen M, Heikkinen E (2003). Relationship between dental health and 10-year mortality in a cohort of community-dwelling elderly people. Eur. J. Oral Sci..

[39] Holm-Pedersen P, Schultz-Larsen K, Christiansen N, Avlund K (2008). Tooth loss and subsequent disability and mortality in old age. J. Am. Geriatr. Soc..

[40] Renvert S, Wallin-Bengtsson V, Berglund J, Persson RG (2015). Periodontitis in older Swedish individuals fails to predict mortality. Clin. Oral Invest..

[41] Moynihan PJ (2007). The relationship between nutrition and systemic and oral well-being in older people. J. Am. Dent. Assoc..

[42] Pihlstrom BL, Michalowicz BS, Johnson NW (2005). Periodontal diseases. Lancet.

[43] Tonetti MS (2017). Dental caries and periodontal diseases in the ageing population: call to action to protect and enhance oral health and well-being as an essential component of healthy ageing – Consensus report of group 4 of the joint EFP/ORCA workshop on the boundaries between caries and periodontal diseases. J. Clin. Periodontol..

[44] Gill B, Harris A, Tredwin C, Gill P (2020). Multimorbidity and oral health: Need for new models of care. Fam. Med. Commun. Health.

[45] Kawamoto M (2021). Relationship between dry mouth and hypertension. Clin. Oral Invest..

[46] Carrizales-Sepúlveda EF, Ordaz-Farías A, Vera-Pineda R, Flores-Ramírez R (2018). Periodontal disease, systemic inflammation and the risk of cardiovascular disease. Heart Lung Circ..

[47] Beck JD, Offenbacher S (2005). Systemic effects of periodontitis: Epidemiology of periodontal disease and cardiovascular disease. J. Periodontol..

[48] Belstrøm D, Damgaard C, Nielsen CH, Holmstrup P (2012). Does a causal relation between cardiovascular disease and periodontitis exist?. Microbes Infect..

[49] Amar S, Wu S-C, Madan M (2009). Is *Porphyromonas gingivalis* cell invasion required for atherogenesis? Pharmacotherapeutic Implications. J. Immunol..

[50] Manabe K, Tanji F, Tomata Y, Zhang S, Tsuji I (2019). Preventive effect of oral self-care on pneumonia death among the elderly with tooth loss: The Ohsaki Cohort 2006 Study. Tohoku J. Exp. Med..

[51] Hayasaka K (2013). Tooth loss and mortality in elderly Japanese adults: Effect of oral care. J. Am. Geriatr. Soc..

[52] Ravald N, Johansson CS (2012). Tooth loss in periodontally treated patients. A long-term study of periodontal disease and root caries. J. Clin. Periodontol..

[53] Müller F, Shimazaki Y, Kahabuka F, Schimmel M (2017). Oral health for an ageing population: The importance of a natural dentition in older adults. Int. Dent. J..

[54] Zhan Y (2014). Prospective study of serum 25-hydroxy vitamin D and tooth loss. J. Dent. Res..

[55] Holter JC (2016). Vitamin D status and long-term mortality in community-acquired pneumonia: Secondary data analysis from a prospective cohort. PLoS ONE.

[56] Ide R (2008). Oral symptoms predict mortality: A prospective study in Japan. J. Dent. Res..

